# Return‐to‐play timing and secondary anterior cruciate ligament injury risk in elite soccer players: A transfermarkt‐based longitudinal analysis

**DOI:** 10.1002/jeo2.70763

**Published:** 2026-05-19

**Authors:** Riccardo D'Ambrosi, Andy Williams, Bertrand Sonnery‐Cottet, Christian Fink, Philipp Baumert

**Affiliations:** ^1^ IRCCS Ospedale Galeazzi – Sant'Ambrogio Milan Italy; ^2^ Dipartimento di Scienze della Vita, della Salute e delle Professioni Sanitarie Università degli Studi “Link Campus University” Rome Italy; ^3^ Fortius Clinic Fitzhardinge Street London UK; ^4^ FIFA Medical Centre of Excellence London UK; ^5^ Centre Orthopédique Santy, FIFA Medical Center of Excellence, Hopital Mermoz, Groupe Ramsay Lyon France; ^6^ Research Unit for Orthopaedic Sports Medicine and Injury Prevention, Institute for Sports Medicine, Alpine Medicine and Health Tourism UMIT TIROL—Private University for Health Sciences and Health Technology Hall Tirol Austria; ^7^ Gelenkpunkt‐Sports and Joint Surgery, FIFA Medical Centre of Excellence Innsbruck/Austria Olympiastraße 39 Innsbruck Austria; ^8^ School of Sport and Exercise Sciences Liverpool John Moores University Liverpool UK

**Keywords:** ACL, ligament, professional athlete, return to play, return to sport, soccer, Transfermarkt

## Abstract

**Purpose:**

Anterior cruciate ligament (ACL) injuries are frequent and potentially career‐threatening in elite soccer. The optimal timing for return‐to‐play (RTP) remains debated, particularly its association with secondary ACL injury. This study evaluated temporal trends in RTP duration and investigated the association between RTP duration, post‐ACL match exposure and secondary ACL injury risk in professional male soccer players.

**Methods:**

Longitudinal publicly available data were analysed from Transfermarkt (2005–2020) on ACL injuries in elite male soccer players from Germany, France, Italy, Spain and the United Kingdom. Players were grouped into three five‐season periods. Multivariable logistic regression was used to examine the association between RTP duration (days) and secondary ACL injury risk, adjusting for time period, post‐ACL match exposure (per 1000 min) and age. Secondary ACL injury proportions and RTP duration trends were compared across time periods.

**Results:**

A total of 1011 ACL injuries were identified in 862 unique players; 15.5% (*n* = 134) sustained ≥2 ACL injuries. Mean RTP duration increased over time (196.7 ± 50.9 days in 2005–2010 vs. 219.9 ± 67.4 days in 2015–2020; *p* < 0.001). The proportion sustaining a secondary ACL injury increased from 9.0% to 16.4% (*p* = 0.031). Post‐ACL match exposure was strongly associated with reinjury risk (OR: 1.39 per 1000 min, *p* < 0.001), and younger age was independently associated with higher risk (OR: 0.86 per year, *p* < 0.001). After adjustment, players in 2010–2020 still exhibited *a* > 2‐fold higher reinjury risk compared with 2005–2010 (*p* < 0.05). Longer RTP duration was not protective and showed a small positive association with reinjury risk (OR: 1.004, *p* = 0.034).

**Conclusion:**

Although RTP duration has increased over the past 15 years in elite soccer, this has not reduced secondary ACL injury risk. Extending RTP duration alone does not appear to reduce reinjury risk.

**Level of Evidence:**

Level III, retrospective cohort study.

**Abbreviations**
ACL: anterior cruciate ligament
ANOVA: analysis of variance
OR: odds ratio
PICOT: population, intervention, comparator, outcome, time
RTP: return‐to‐play

## INTRODUCTION

Anterior cruciate ligament (ACL) injuries represent one of the most severe and career‐threatening conditions in elite soccer players. They are associated with long absences from competition, reduced performance after return and a substantial risk of reinjury, particularly in younger athletes and those involved in high‐demand pivoting sports like soccer [6, 28, 36]. Although surgical reconstruction and structured rehabilitation are the standard of care, reinjury rates remain highly variable, with estimates ranging from 2% to 25% in elite athletes [13, 14, 18, 32, 37].

The concept of delaying return‐to‐play (RTP) beyond 9 months postoperatively has gained traction as a potential strategy to reduce reinjury risk. Evidence from prospective cohorts suggests that athletes who return earlier face significantly higher odds of sustaining a second ACL injury [4, 5, 17]. As a result, clinical guidelines now increasingly recommend a combination of time‐based and functional criteria before clearance to return [24]. However, the extent to which these recommendations have been adopted in elite‐level soccer players, and whether they have led to meaningful changes in reinjury rates over time, remains unclear.

Fear of reinjury, reduced confidence and performance anxiety are prevalent among ACL‐reconstructed athletes and may independently influence both RTP timing and reinjury risk [2, 8].

Despite the growing emphasis on evidence‐based RTP protocols, longitudinal real‐world data examining trends in RTP timing and reinjury risk in elite soccer players are lacking. Moreover, it is unknown whether increased RTP durations at the population level have had a protective effect in reducing second ACL injuries.

The primary aim of this study was to evaluate temporal trends in RTP following ACL injury among elite male soccer players between 2005 and 2020. The secondary objective was to assess the association between RTP duration and the risk of secondary ACL injury.

We hypothesised that (1) RTP duration has increased over time and (2) longer RTP duration would be associated with a lower risk of reinjury.

According to the PICOT framework, the population (P) comprised elite male soccer players from the top five European leagues; the exposure (I) was RTP duration following ACL injury; the comparator (C) was variation across RTP durations and time periods; the outcome (O) was secondary ACL injury; and the time frame (T) included injuries sustained between 2005 and 2020 with follow‐up extending up to three subsequent seasons [30].

## MATERIAL AND METHODS

This was a retrospective cohort study analysing publicly available data from *Transfermarkt.co.uk/*, a widely used online database that tracks injuries, players' performance statistics and match appearances across professional soccer players. Only male players were included because previous reliability assessments of Transfermarkt injury data have predominantly focused on male professional football [19, 23]. The dataset included ACL injuries sustained by male players in the first and second divisions of the top five European leagues (Germany, France, Italy, Spain and the United Kingdom) between the 2005/2006 and 2019/2020 seasons. While the primary analysis focused on these leagues, players who transferred to other competitions during follow‐up remained in the dataset; therefore, additional leagues appear in the club‐level dataset (Table S1). To ensure a consistent follow‐up window of up to three subsequent seasons for all players, reinjury surveillance data were extracted through the 2023/2024 season. The dataset included dates of injury onset and RTP. According to Grassi et al., RTP was defined as return to team training, calculated based on the number of days absent from sport, as adopted from transfermarkt [15].

ACL injuries with implausibly short recovery durations (<90 days) or return dates beyond 400 days (equivalent to approximately 13 months) were excluded to remove likely data errors. Based on these criteria, 74 injuries were excluded from the dataset prior to analysis.

Transfermarkt data have been previously evaluated for epidemiological research in professional soccer players and, for severe injuries such as ACL ruptures, provide reasonably reliable estimates of injury dates and return to play timing based on reported time loss and subsequent official match participation [15, 26, 29]. Although Transfermarkt is a publicly available, community‐driven database and its use may introduce reporting bias and incomplete injury capture, previous studies have demonstrated that severe injuries such as ACL ruptures are reported with acceptable validity in professional soccer due to their clinical and media relevance [23]. The TITAN checklist was fulfilled to transparently report the use of artificial intelligence [1].

### Injury definition and inclusion criteria

Players were included if they had a documented ACL injury confirmed by Transfermarkt records. Only players with clearly reported dates of injury and RTP were included. Goalkeepers were excluded to limit exposure‐related variability. For the purpose of reinjury analysis, only players with a first ACL injury occurring between the 2005/2006 and 2019/2020 seasons were considered to ensure sufficient follow‐up time to detect a subsequent ACL injury.

ACL reinjury was defined as any additional ACL injury (ipsilateral or contralateral) reported after the player had returned to official match play. For each player with an initial ACL injury, data were analysed for up to three consecutive seasons. The data did not distinguish between surgical revision and new injury, nor between ipsilateral and contralateral tears.

### Variables and outcomes

The primary exposure variable was RTP duration, defined as the number of days between the date of first ACL injury and the date of RTP. The primary outcome was ACL reinjury (yes/no).

Players were grouped into three five‐season periods to evaluate longitudinal trends:

- Period 1: 2005/2006–2009/2010
- Period 2: 2010/2011–2014/2015
- Period 3: 2015/2016–2019/2020

This grouping was chosen to enable the analysis of temporal trends while maintaining adequate sample size within each period and minimising the impact of year‐to‐year variability [31].

Post‐ACL match exposure was quantified as the cumulative number of official match minutes played after RTP during the observation period and was normalised per 1000 min.

Age at the time of the first ACL injury was calculated from birthdate and injury date and included as a continuous covariate. Additional descriptive subgroup analyses were conducted based on age categories (<24, 24–30, >30 years) and playing position (central defences, lateral defences, central midfields, lateral midfields, forwards).

### Statistical analysis

Descriptive statistics were calculated for RTP durations (mean, median, standard deviation) and secondary ACL injury outcomes across the three time periods. Normality of RTP distribution was assessed using visual inspection of histograms and Q–Q plots and the Shapiro–Wilk test. Between‐period differences in RTP duration were analysed using one‐way analysis of variance (ANOVA) or the Kruskal–Wallis test, as appropriate, with Tukey or Dunn post hoc tests for pairwise comparisons. Differences in the proportion of players sustaining a secondary ACL injury across time periods were assessed using chi‐square tests. To examine the association between RTP duration and secondary ACL injury risk, a multivariable logistic regression analysis was performed with secondary ACL injury (yes/no) as the binary dependent variable. RTP duration was included as a continuous predictor and models were adjusted for time period, post‐ACL match exposure, and age at injury. Effect sizes were reported as odds ratios (ORs) with 95% confidence intervals (CIs), calculated by exponentiating the regression coefficients. Interaction terms between RTP duration and time period were additionally tested to assess potential temporal effect modification. Statistical significance was defined as *p* < 0.05. All analyses were conducted in Python (version 3.11.13) using Google Colaboratory, employing packages including pandas, numpy, statsmodels and scipy.

## RESULTS

On average, each injured player had 2.8 ± 1.3 seasons recorded after their ACL injury. A total of 1011 ACL injuries were identified among 862 individual elite male soccer players sustaining a first ACL injury between the 2005/2006 and 2019/2020 seasons in the first and second divisions of the top five European leagues. Baseline characteristics of the cohort stratified by time period are presented in Table 1.

**Table 1:** Baseline characteristics of players with first ACL injury.

Variable	Total (*n* = 862)	2005–2010 (*n* = 166)	2010–2015 (*n* = 318)	2015–2020 (*n* = 378)
ACL injuries (*n*)	1011	183	384	444
Age at injury, years (mean ± SD)	25.2 ± 4.1	25.2 ± 4.1	25.3 ± 4.2	25.0 ± 4.1
Age <24 years, *n* (%)	379 (44.0)	73 (44.0)	132 (41.5)	174 (46.0)
Age 24–30 years, *n* (%)	359 (41.6)	66 (39.8)	137 (43.1)	156 (41.3)
Age >30 years, *n* (%)	124 (14.4)	27 (16.3)	49 (15.4)	48 (12.7)
Central defences, *n* (%)	192 (22.3)	33 (19.9)	82 (25.8)	77 (20.4)
Lateral defences, *n* (%)	140 (16.2)	32 (19.3)	43 (13.5)	65 (17.2)
Central midfields, *n* (%)	235 (27.3)	47 (28.3)	83 (26.1)	105 (27.8)
Lateral midfields, *n* (%)	130 (15.1)	20 (12.0)	48 (15.1)	62 (16.4)
Forwards, *n* (%)	165 (19.1)	34 (20.5)	62 (19.5)	69 (18.3)

Abbreviations: ACL, anterior cruciate ligament; SD, standard deviation.

**Figure 1:**
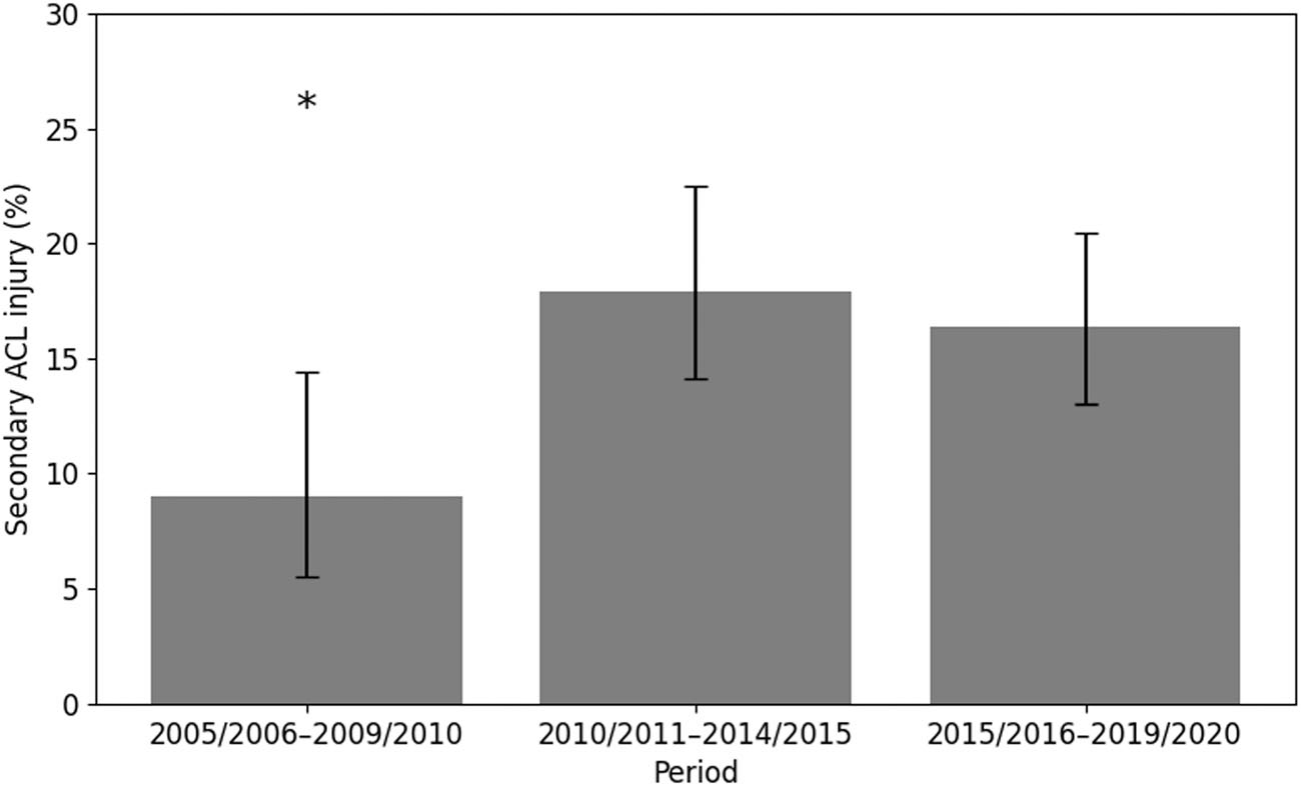
Secondary ACL injury proportion by period. Error bars represent 95% Wilson confidence intervals. * Indicates that the 2005/2006–2009/2010 period differs significantly from both other periods (*p* < 0.001). ACL, anterior cruciate ligament.

Lower numbers in the earliest and most recent seasons likely reflect edge effects of the observational window. Fewer players were captured at the beginning of the dataset, and shorter follow‐up was available for the most recent years, a limitation commonly reported in studies using publicly available injury databases [19, 23]. To ensure a consistent follow‐up of up to three subsequent seasons for all players, reinjury surveillance data were extracted through the 2023/2024 season. Among these, 728 players sustained only one ACL injury, whereas 134 players (15.5%) suffered two or more ACL injuries, either ipsilateral or contralateral. The age at first ACL injury remained consistent across the three time periods, with a mean of 25.2 ± 4.1 years (*p* = 0.55). However, the proportion of players sustaining a second ACL injury increased significantly from 9.0% in 2005–2010 to 17.9% in 2010‐15, and then remained at a similar level at 16.4% in 2015–2020 (*p* = 0.031; Figure 1).

Table 2 presents ACL injury events (*n* = 1011) stratified by time period and used for the analysis of RTP duration and secondary ACL injury rates. The average RTP over all three periods was 209.8 ± 60.5 days.

**Table 2:** Return‐to‐play duration and secondary ACL injury by period.

Period	*n*	RTP mean + SD (days)	Secondary ACL injury (%)
2005/06–2009/10	183	196.7 ± 50.9	9.0*
2010/11–2014/15	384	204.4 ± 54.1	17.9
2015/16–2019/20	444	219.9 ± 67.4*	16.4

Abbreviations: ACL, anterior cruciate ligament; RTP, return‐to‐play; SD, standard deviation.

* Statistically different to both other time periods, *p* < 0.05.

**Figure 2:**
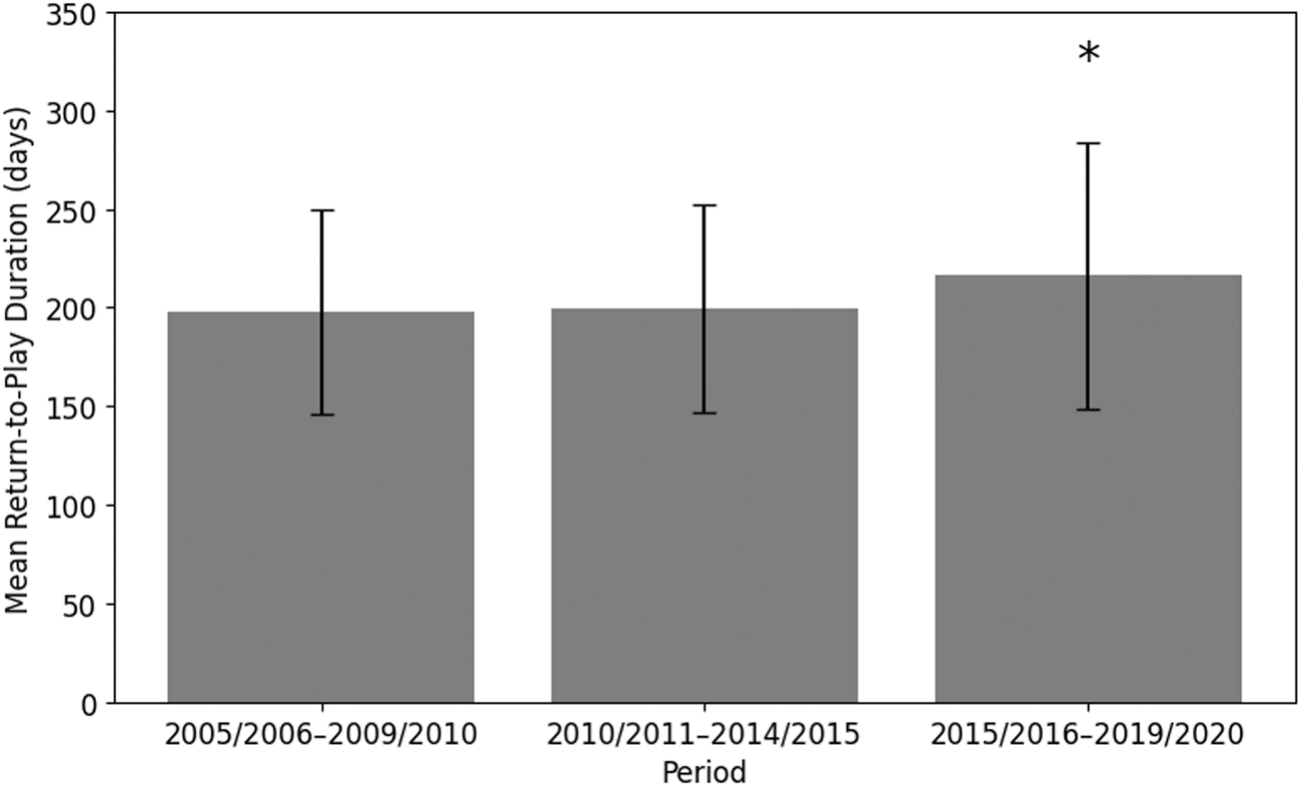
Mean return‐to‐play duration over time. Data are presented as mean ± standard deviation. * Indicates that the 2015/2016–2019/2020 period differs significantly from both earlier periods (*p* < 0.001).

There was a progressive increase in mean RTP duration from 196.7 ± 50.9 days in 2005–2010 to 219.9 ± 67.4 days in 2015–2020, with a significant overall difference between periods (*p* < 0.001). As illustrated in Figure 2, post hoc comparisons showed that RTP duration in 2015/2016–2019/2020 was significantly longer than in both earlier periods (*p* < 0.001).

Table 3 shows detailed annual incidence of ACL injuries and re‐injuries.

**Table 3:** Annual distribution of secondary ACL injuries by year of first ACL injury.

First ACL year	Number of players	Number with ≥2 ACL injuries	Secondary ACL injury (%)
2005/2006	5	1	20
2006/2007	9	0	0
2007/2008	23	2	8
2008/2009	48	4	8
2009/2010	58	6	10
2010/2011	50	8	16
2011/2012	53	8	15
2012/2013	63	13	21
2013/2014	71	14	20
2014/2015	75	8	10
2015/2016	60	16	27
2016/2017	70	12	17
2017/2018	77	17	22
2018/2019	75	9	12
2019/2020	93	12	13
2020	32	4	16

Abbreviation: ACL, anterior cruciate ligament.

Multivariable logistic regression was performed to identify factors associated with reinjury risk. In contrast to the unadjusted observations, the multivariable model adjusting for time period, match exposure and age revealed a very small but statistically significant positive association between RTP duration and reinjury risk (*β* = 0.0036, *p* = 0.034), corresponding to a 0.4% increase in the odds of reinjury per additional day of RTP duration (OR: 1.004, 95% CI: 1.001–1.007).

Consistent with this, no significant interaction effects between RTP duration and time period on reinjury risk were found (2010–2015: *p* = 0.460; 2015–2020: *p* = 0.764), indicating that the association between RTP duration and reinjury risk was stable across all time periods.

Post‐ACL match exposure emerged as the strongest predictor of reinjury risk. Each additional 1000 min of post‐ACL match exposure was associated with a 38.5% increase in the odds of a second ACL injury (OR: 1.39, 95% CI: 1.28–1.51; *p* < 0.001). After adjustment for RTP duration, post‐ACL exposure and age, players in the 2010–2015 and 2015–2020 periods showed significantly higher odds of reinjury compared with the 2005–2010 reference period (OR: 2.35 [95% CI: 1.21–4.57] and OR: 2.17 [95% CI: 1.13–4.16], respectively).

Age at the time of the first ACL injury was independently associated with reinjury risk. Players aged 24–30 had a significantly lower risk compared to those under 24 years (OR: 0.56, 95% CI: 0.38–0.83; *p* = 0.004), while players older than 30 had an even greater reduction in risk (OR: 0.12, 95% CI: 0.04–0.33; *p* < 0.001). Playing position was not significantly associated with reinjury risk (all *p* > 0.45).

**Figure 3:**
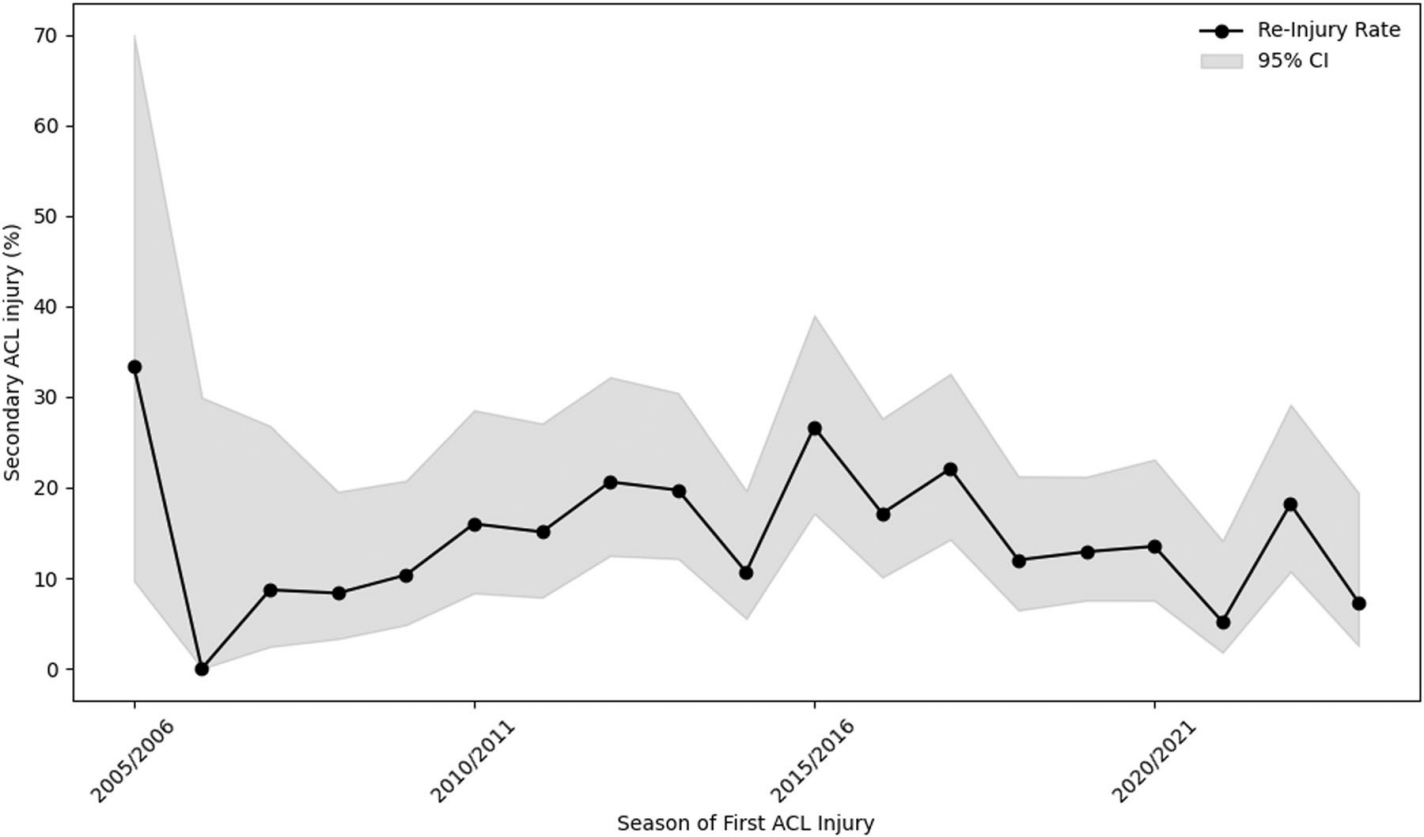
Secondary ACL injury rate by season of first ACL injury (2005–2023). Shaded areas represent 95% CI. ACL, anterior cruciate ligament; CI, confidence intervals.

Annual reinjury rates fluctuated over the study period, with a peak observed in 2015 (27%, 16 of 60 players). Figure 3 illustrates the annual variation in reinjury rates from 2005 to 2023. Years at the beginning and end of the dataset (e.g., 2005 and 2023) had smaller sample sizes and should be interpreted with caution.

**Figure 4:**
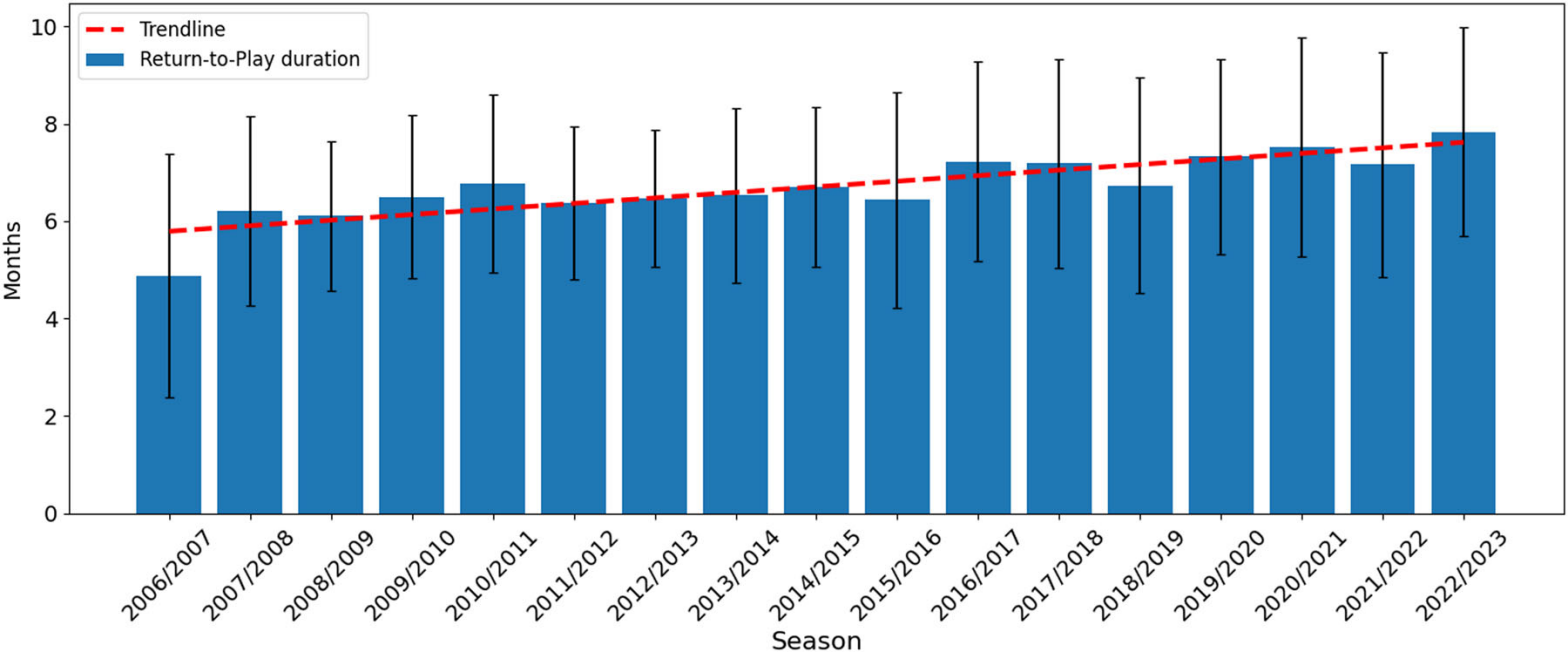
Temporal distribution of secondary ACL injuries. Data are presented as mean ± standard deviation. ACL, anterior cruciate ligament.

The distribution of RTP times showed a rightward shift over time, indicating a growing tendency toward longer rehabilitation (Figure 4).

**Figure 5:**
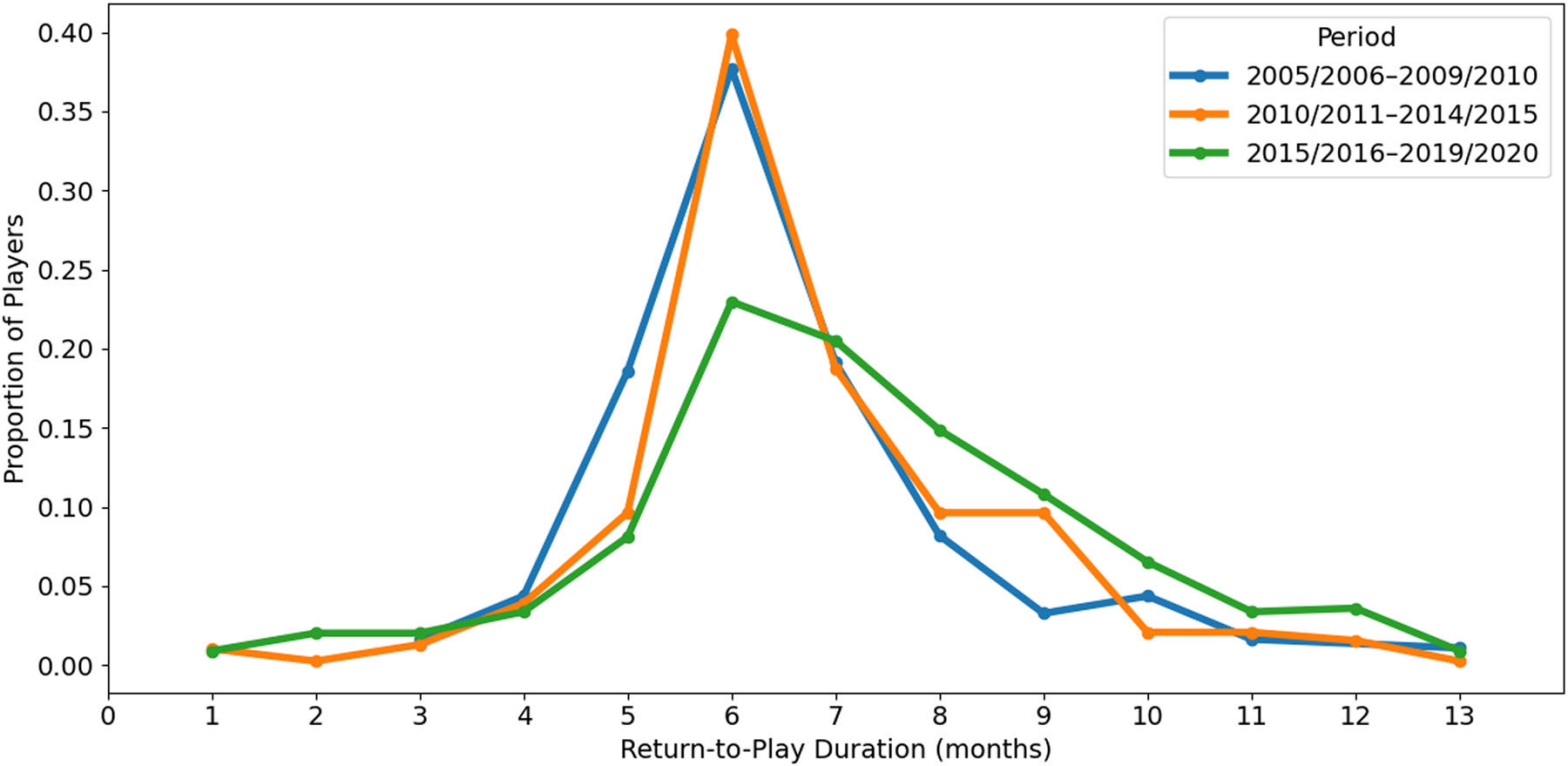
Distribution of return‐to‐play duration (in months) across study periods. Lines represent the proportion of players returning to sport at each month after ACL injury. ACL, anterior cruciate ligament.

The overall peak in RTP occurred by 6 months postinjury, with very few players returning before 4–5 months. Figure 5 shows the normalised distribution of RTP durations across the three study periods, reflecting this gradual change in recovery patterns.

## DISCUSSION

The most important finding of this study is that longer RTP duration was not associated with a reduced risk of secondary ACL injury in elite soccer players. Although RTP duration increased significantly over time, this was not accompanied by a reduction in reinjury rates.

Accordingly, the first hypothesis, that RTP duration has increased over time, was confirmed, whereas the second hypothesis, that longer RTP duration would be associated with a lower risk of reinjury, was not supported.

These findings challenge the common assumption that simply delaying RTP confers protection against secondary ACL injury and suggest that time‐based criteria alone may be insufficient to mitigate reinjury risk in elite soccer players.

Longer RTP duration showed a statistically significant but very small association with reinjury risk (*β* = 0.0036), indicating a negligible increase in the odds of reinjury per additional day.

No significant interaction between RTP duration and time period was observed, indicating that the relationship between RTP duration and reinjury risk was consistent across different time periods. Overall, longer RTP duration was not associated with a reduced risk of secondary ACL injury in elite soccer players.

The observed increase in secondary ACL injury rates between the early and middle study periods, followed by a relative stabilisation in the most recent period, should be interpreted with caution. This pattern may partly reflect improvements in injury reporting and data capture over time in publicly available datasets, as previously described [19, 23]. In addition, evolving physical demands in elite soccer, including increased match intensity and fixture congestion, may have contributed to higher reinjury risk in more recent years [11, 27]. The subsequent plateau may suggest a stabilisation of these factors or a potential ceiling effect in reinjury risk despite changes in rehabilitation strategies.

Previous research has shown that returning to high‐risk pivoting sports before 9 months postoperatively is associated with increased reinjury risk, particularly among younger athletes [4, 5, 17]. Further, Nagelli and Hewett reported that delaying return to high‐level activity until 2 years after ACL reconstruction significantly reduces the risk of a second ACL injury [25]. However, more recent studies have questioned the generalisability of fixed, time‐based criteria [4, 5, 7, 17, 35]. Furthermore, such long recoveries would simply not be accepted in professional soccer players.

Kotsifaki et al. recently investigated the status of male athletes 2 years after ACL reconstruction, the factors affecting a return to pivoting sports, and the association between time to RTP and subsequent knee injury risk for those athletes who met discharge criteria. Completing rehabilitation and meeting objective criteria significantly increased the odds for male athletes to return to pivoting sports. Time to RTP did not impact the risk for a new knee or ACL injury if athletes met objective criteria [21, 22].

While average RTP duration has increased over time, this temporal change was not accompanied by a reduction in reinjury rates. However, this finding should be interpreted with caution, as RTP duration alone may not adequately reflect true biological, functional or psychological readiness for return to sport. Therefore, the observed lack of association may partly reflect limitations in using time‐based metrics as a surrogate for recovery.

Another potential explanation for the observed increase in RTP duration over time is the evolution of diagnostic and surgical approaches. Advances in imaging techniques, particularly magnetic resonance imaging and increased awareness of concomitant injuries, such as meniscal ramp lesions, have likely improved the detection of associated pathologies. This may have led to more comprehensive treatment strategies, which in turn could contribute to longer rehabilitation timelines and delayed return to play [38].

The lack of a protective association between longer RTP duration and reinjury risk observed in this study may reflect the limitations of using time alone as a surrogate for recovery. Indeed, objective functional criteria, such as strength symmetry, hop performance and movement quality, have been shown to better reflect readiness for return to sport [10, 16, 20, 21, 22, 23]. However, such measures are not available in large‐scale observational datasets like the present one. In addition, RTP decisions in elite soccer are influenced by external factors, including competitive demands and contextual pressures, which may further limit the relevance of time‐based criteria alone [34].

Psychological factors, such as fear of reinjury and confidence, may influence RTP decisions and outcomes. However, these variables were not available in the present dataset and therefore could not be evaluated, representing an additional limitation when interpreting reinjury risk [12, 33].

A recent systematic review and meta‐analysis focusing exclusively on professional soccer players reported high RTP rates after ACL reconstruction, with more than 92% of athletes returning to competition and approximately 80% regaining their pre‐injury level. However, a key temporal finding was a significant prolongation of time to RTP in more recent cohorts, exceeding 9 months in studies published after 2021, without a corresponding reduction in graft re‐rupture rates, which remained stable at approximately 8% [9].

Post‐ACL match exposure emerged as the strongest predictor of secondary ACL injury in this cohort. Each additional 1000 min of postreturn match exposure was associated with a substantial increase in reinjury odds, underscoring the central role of cumulative mechanical load after RTP. Notably, even after adjustment for exposure, RTP duration and age, players in the more recent periods exhibited a more than twofold higher reinjury risk compared with the earliest period.

These findings should be interpreted in the context of evolving demands in elite soccer. Over the past decade, players have been exposed to increasing match frequency, fixture congestion and reduced recovery time between games, all of which have been associated with a higher injury risk [11, 27]. This changing competitive environment may contribute to the persistence of elevated reinjury risk despite longer rehabilitation timelines.

Age at first ACL injury was independently associated with reinjury risk, with younger players demonstrating a higher susceptibility to secondary ACL injury. This finding is consistent with previous literature reporting higher reinjury rates in younger athletes [6, 14, 37]. These results suggest that age should be considered when evaluating RTP strategies and reinjury risk.

Reinjury rates in elite soccer players have remained relatively consistent across studies despite advances in surgical techniques [3, 6, 13, 18, 28, 32]. However, the present study was not designed to evaluate the underlying mechanisms of this observation, and such interpretations should be made with caution.

One possible explanation is that the external demands of elite soccer have increased over time, including greater match frequency, fixture congestion and reduced recovery time between games. These factors have been shown to be associated with a higher risk of injury in professional players and may contribute to the persistence of reinjury risk despite longer rehabilitation timelines [11, 27].

If players now return to a more demanding competitive environment than a decade ago, similar RTP durations may carry higher biomechanical and neuromuscular loads, effectively increasing reinjury risk despite unchanged timeframes. This evolving context underscores the limitations of calendar‐based approaches and the need to assess readiness in light of sport‐specific demands [27].

The observed peak in reinjury rates in 2015 (27%) should be interpreted with caution, as year‐to‐year variations may be influenced by relatively small sample sizes and inherent variability in observational datasets. In addition, the definition of secondary ACL injury in the present study included both ipsilateral and contralateral injuries, and therefore does not specifically reflect graft failure rates. This represents an important limitation when interpreting temporal fluctuations in reinjury rates [15, 36].

These findings suggest that time‐based RTP thresholds alone may be insufficient to reduce reinjury risk in elite soccer players. A more comprehensive approach to RTP decision‐making may therefore be required.

### Limitations

This study has several important limitations that should be considered when interpreting the findings. The use of publicly available data (Transfermarkt) limits access to key clinical variables, including graft type, surgical technique, rehabilitation protocols and reinjury laterality. Consequently, subsequent ACL injuries could not be classified as ipsilateral graft failures or contralateral injuries and were therefore analysed collectively as secondary ACL injuries, which may limit the specificity of the outcome.

The RTP definition used in this study, based on return to match availability, may not accurately reflect functional or biological readiness and does not account for performance level at return. In addition, several potentially relevant confounders, including psychological status, training exposure, match load and prior injury history, were not available, resulting in potential residual confounding.

Importantly, reliance on publicly obtained, community‐driven datasets introduce additional sources of reporting bias and potential underestimation of injury events, particularly among less prominent players [19].

Furthermore, regression models did not include certain contextual variables, such as team medical policies, surgeon variability or club‐level injury management strategies, which may also influence outcomes. Subgroup analyses should be considered exploratory due to limited statistical power.

Finally, the mean follow‐up duration of approximately 2.8 seasons may not be sufficient to capture all secondary ACL injuries, particularly those occurring in the longer term. Although most reinjuries are reported within the first 2–3 years after return to play [25], later events may have been missed, potentially leading to an underestimation of overall reinjury risk [17, 19].

Taken together, these limitations reflect the observational nature of the study and warrant cautious interpretation of the findings.

## CONCLUSION

RTP duration following ACL injury has increased over time among elite soccer players, without a corresponding reduction in the rate of secondary ACL injury. Longer RTP duration was not associated with a lower risk of reinjury, whereas post‐ACL match exposure and younger age were identified as significant risk factors. These findings suggest that delaying return to play alone may be insufficient to reduce reinjury risk in elite soccer players.

## AUTHOR CONTRIBUTIONS

**Riccardo D'Ambrosi**: Conceptualisation; writing—original draft preparation. **Andy Williams**: Clinical interpretation; writing—review and editing. **Bertrand Sonnery‐Cottet**: Clinical interpretation; writing—review and editing. **Christian Fink**: Conceptualisation; clinical interpretation; writing—review and editing. **Philipp Baumert**: Conceptualisation; methodology; statistical analysis; writing—review and editing. All authors contributed to data interpretation, critically revised the manuscript for important intellectual content, approved the final version of the manuscript, and agree to be accountable for all aspects of the work.

## CONFLICT OF INTEREST STATEMENT

The authors declare no conflicts of interest.

## ETHICS STATEMENT

All authors consent to the publication of the manuscript.

## Supporting information

Supporting File 1

Supporting File 2

Supporting File 3

## Data Availability

Raw data are available upon request to the corresponding author.
