# Supplementary material for: Return‐to‐play timing and secondary anterior cruciate ligament injury risk in elite soccer players: A transfermarkt‐based longitudinal analysis
Source: J Exp Orthop. 2026 May 19;13(2):e70763. doi: 10.1002/jeo2.70763 (PMC13185221; doi:10.1002/jeo2.70763)
Supplement: Supplementary file 1 — Supporting File 1 [file JEO2-13-e70763-s003.docx]

STROBE Statement—checklist of items that should be included in reports of observational studies

|  | Item No. | Recommendation | Page  No. | Relevant text from manuscript |
| --- | --- | --- | --- | --- |
| **Title and abstract** | 1 | (*a*) Indicate the study’s design with a commonly used term in the title or the abstract | Title Page | “Study Design: Retrospective Cohort Study, Level of Evidence: 3” |
|  |  | (*b*) Provide in the abstract an informative and balanced summary of what was done and what was found | Title Page | Abstract contains Background, Purpose, Methods, Results, Conclusion |
| Introduction | | | |  |
| Background/rationale | 2 | Explain the scientific background and rationale for the investigation being reported | 3-4 | “ACL injuries… optimal timing for return to sport remains debated… limited longitudinal evidence.” |
| Objectives | 3 | State specific objectives, including any prespecified hypotheses | 4 | “The primary aim of this study was to evaluate temporal trends in RTS duration… secondary objective was to investigate whether longer RTS durations were associated with reduced risk.” |
| Methods | | | |  |
| Study design | 4 | Present key elements of study design early in the paper | 4-5 | “This was a retrospective cohort study analysing publicly available data…” |
| Setting | 5 | Describe the setting, locations, and relevant dates, including periods of recruitment, exposure, follow-up, and data collection | 4-5 | “Dataset included ACL injuries… in the first and second divisions of the top five European leagues… 2005/2006 to 2019/2020 seasons.” |
| Participants | 6 | (*a*) *Cohort study*—Give the eligibility criteria, and the sources and methods of selection of participants. Describe methods of follow-up  *Case-control study*—Give the eligibility criteria, and the sources and methods of case ascertainment and control selection. Give the rationale for the choice of cases and controls  *Cross-sectional study*—Give the eligibility criteria, and the sources and methods of selection of participants | 4-5 | \|  \| \| --- \| \| “Players included if they had a documented ACL injury… clearly reported dates of injury and return… goalkeepers excluded.” \| \| |
|  |  | (*b*) *Cohort study*—For matched studies, give matching criteria and number of exposed and unexposed  *Case-control study*—For matched studies, give matching criteria and the number of controls per case |  |  |
| Variables | 7 | Clearly define all outcomes, exposures, predictors, potential confounders, and effect modifiers. Give diagnostic criteria, if applicable | 5 | “Primary exposure: RTS duration (days)… primary outcome: ACL reinjury (yes/no)… subgroup variables: age, position.” |
| Data sources/ measurement | 8* | For each variable of interest, give sources of data and details of methods of assessment (measurement). Describe comparability of assessment methods if there is more than one group | *5* | *“Data from Transfermarkt.co.uk, previously validated… RTS defined as first official match appearance after injury.”* |
| Bias | 9 | Describe any efforts to address potential sources of bias | 4-5, 14 | “Implausible data (<90 or >400 days) excluded… limitations section discusses potential selection and information bias due to public database.” |
| Study size | 10 | Explain how the study size was arrived at | 6 | “A total of 1011 ACL injuries identified among 862 players… numbers determined by inclusion of all eligible cases in dataset.” |

Continued on next page

| Quantitative variables | 11 | Explain how quantitative variables were handled in the analyses. If applicable, describe which groupings were chosen and why | 5–6 | “RTS duration analysed as continuous variable in logistic regression; grouped into 5-year periods for trend analysis.” |
| --- | --- | --- | --- | --- |
| Statistical methods | 12 | (*a*) Describe all statistical methods, including those used to control for confounding | 5-6 | \| „ \| \| --- \| \| ANOVA/Kruskal–Wallis for group comparisons; logistic regression for association between RTS and reinjury risk.” \| \| |
|  |  | (*b*) Describe any methods used to examine subgroups and interactions | 6 | “Analyses stratified by age and position; interaction tested between RTS duration and time period.” |
|  |  | (*c*) Explain how missing data were addressed | 5-6 | “Cases with implausible or missing RTS dates excluded; no imputation performed.” |
|  |  | (*d*) *Cohort study*—If applicable, explain how loss to follow-up was addressed  *Case-control study*—If applicable, explain how matching of cases and controls was addressed  *Cross-sectional study*—If applicable, describe analytical methods taking account of sampling strategy |  |  |
|  |  | (*e*) Describe any sensitivity analyses |  |  |
| Results | | | | |
| Participants | 13* | (a) Report numbers of individuals at each stage of study—eg numbers potentially eligible, examined for eligibility, confirmed eligible, included in the study, completing follow-up, and analysed | 6 | A total of 1011 ACL injuries among 862 players were identified and included.” |
|  |  | (b) Give reasons for non-participation at each stage | 5-6 | \|  \| \| --- \| \| “Cases with implausible RTS duration (<90 or >400 days) or incomplete data were excluded (n=32).” \| \| |
|  |  | (c) Consider use of a flow diagram | - | Not applicable; numbers fully reported in text. |
| Descriptive data | 14* | (a) Give characteristics of study participants (eg demographic, clinical, social) and information on exposures and potential confounders | 6-10 | \|  \| \| --- \|  \| “Mean (±SD) age 25.6 ± 4.1 years; majority midfielders (41%).” \| \| --- \| |
|  |  | (b) Indicate number of participants with missing data for each variable of interest | 6 | “After exclusions, no missing data remained for primary outcomes.” |
|  |  | (c) *Cohort study*—Summarise follow-up time (eg, average and total amount) | 6 |  |
| Outcome data | 15* | *Cohort study*—Report numbers of outcome events or summary measures over time | *6-7* | *“Median RTS duration 248 days (IQR 212–280). Overall reinjury rate 17.8%.”* |
|  |  | *Case-control study—*Report numbers in each exposure category, or summary measures of exposure |  |  |
|  |  | *Cross-sectional study—*Report numbers of outcome events or summary measures |  |  |
| Main results | 16 | (*a*) Give unadjusted estimates and, if applicable, confounder-adjusted estimates and their precision (eg, 95% confidence interval). Make clear which confounders were adjusted for and why they were included | 7-8 | \|  \| \| --- \| \| “Logistic regression: longer RTS duration not significantly associated with reduced reinjury risk (OR 0.99, 95% CI 0.97–1.01).” \| \| |
|  |  | (*b*) Report category boundaries when continuous variables were categorized | 7 | “RTS grouped by 5-year blocks (2005–2010, 2011–2015, 2016–2020). |
|  |  | (*c*) If relevant, consider translating estimates of relative risk into absolute risk for a meaningful time period | 7-8 | “Reinjury risk remained ~18% across time periods despite longer RTS.” |

Continued on next page

| Other analyses | 17 | Report other analyses done—eg analyses of subgroups and interactions, and sensitivity analyses | 8 | \|  \| \| --- \| \| “Subgroup analyses by age group and position showed consistent results.” \| \| |
| --- | --- | --- | --- | --- | --- | --- | --- |
| Discussion | | | | |
| Key results | 18 | Summarise key results with reference to study objectives | 11 | “RTS durations increased substantially over 15 years, but reinjury risk remained unchanged.” |
| Limitations | 19 | Discuss limitations of the study, taking into account sources of potential bias or imprecision. Discuss both direction and magnitude of any potential bias | 14 | \|  \| \| --- \| \| “Potential bias due to publicly available data… possible underreporting of reinjuries… lack of clinical verification.” \| \| |
| Interpretation | 20 | Give a cautious overall interpretation of results considering objectives, limitations, multiplicity of analyses, results from similar studies, and other relevant evidence | 9-14 | “Findings challenge the assumption that longer time alone reduces reinjury risk… supports need for multifactorial RTS decision-making.” |
| Generalisability | 21 | Discuss the generalisability (external validity) of the study results | 13-14 | “Results generalisable to elite male football players in top European leagues; not necessarily applicable to other populations.” |
| Other information | |  | | |
| Funding | 22 | Give the source of funding and the role of the funders for the present study and, if applicable, for the original study on which the present article is based | 14 | “No specific funding was received for this study. Authors declare no conflicts of interest.” |

*Give information separately for cases and controls in case-control studies and, if applicable, for exposed and unexposed groups in cohort and cross-sectional studies.

**Note:** An Explanation and Elaboration article discusses each checklist item and gives methodological background and published examples of transparent reporting. The STROBE checklist is best used in conjunction with this article (freely available on the Web sites of PLoS Medicine at http://www.plosmedicine.org/, Annals of Internal Medicine at http://www.annals.org/, and Epidemiology at http://www.epidem.com/). Information on the STROBE Initiative is available at www.strobe-statement.org.
