# Supplementary material for: Return‐to‐play timing and secondary anterior cruciate ligament injury risk in elite soccer players: A transfermarkt‐based longitudinal analysis
Source: J Exp Orthop. 2026 May 19;13(2):e70763. doi: 10.1002/jeo2.70763 (PMC13185221; doi:10.1002/jeo2.70763)
Supplement: Supplementary file 3 — Supporting File 3 [file JEO2-13-e70763-s001.docx]

| **TITAN Guideline Checklist 2025** | | | |
| --- | --- | --- | --- |
| **Topic** | **Item** | **Description** | **Page number** |
| **Artificial Intelligence (AI) (some journals may prefer this in the methods and/or acknowledgments section and it should also be declared in the cover letter)** | 1 | **Declaration of whether any AI was used in the research and manuscript development**  **State no, if that’s the case.**  **If yes, proceed to item 1a.** | p. 14, Declarations: “Artificial intelligence tools were used in this work…” |
|  | 1a | **Purpose and Scope of AI Use**  - Precisely state why AI was employed (e.g. development of research questions, language drafting, statistical analysis/summarisation, image annotation, etc).  - Was generative AI utilised and if so, how?  - Clarify the stage(s) of the reporting workflow affected (planning, writing, revisions, figure creation). - Confirmation that the author(s) take responsibility for the integrity of the content affected/generated | p. 14, Declarations – used for language polishing of manuscript drafts and for drafting code snippets in Python to structure analysis (fully reviewed and verified by authors) |
|  | 1b | **AI Tool(s) and Configuration**  - Name each system (vendor, model, major version/date).  - State the date it was used  - Specify relevant parameters (e.g. prompt length, plug-ins, fine-tuning, temperature).  - Declare whether the tool operated locally on-premises, or via a cloud API and any integrations with other systems. | p. 14: OpenAI ChatGPT (ChatGPT, GPT- 4o, May-July 2025, cloud API). Used with simple text prompts for code and language support. No fine-tuning, plug-ins, or integrations applied. |
|  | 1c | **Data Inputs and Safeguards**  - Describe categories of data provided to the AI (patient text, de-identified images, literature abstracts).  - Confirm that all inputs were de-identified and compliant with GDPR/HIPAA.  - Note any institutional approvals or data-sharing agreements obtained. | p. 14, Declarations: Only publicly available datasets (Transfermarkt) and non-sensitive draft text were entered. No patient or confidential data used. |
|  | 1d | **Human Oversight and Verification**  - Identify the supervising author(s) who reviewed every AI output.  - Detail the process for fact-checking, clinical accuracy checks  - State whether any AI-generated text/figures were edited or discarded. - Acknowledge the limitations of AI and its use | p. 14, Declarations: All outputs reviewed and verified by PB (corresponding author). Draft code tested and revised before use. No unverified AI output included. |
|  | 1e | **Bias, Ethics and Regulatory Compliance**  - Outline steps taken to detect and mitigate algorithmic bias (e.g. cross-checking against under-represented populations).  - Affirm adherence to relevant ethical frameworks.  - Disclose any conflicts of interest or financial ties to AI vendors. | p. 14: Minimal bias risk as no patient-level or clinical data involved. Compliance with ethical standards and GDPR ensured. No conflicts of interest with AI vendors. |
|  | 1f | **Reproducibility and Transparency**  - Provide the exact prompts or code snippets (as supplementary material if lengthy).  - Supply version-controlled logs or model cards where possible.  - if applicable, state repository, hyperlink or digital object identifier (DOI) where AI-generated artefacts can be accessed, enabling attempts at independent replication of the query/input. | p. 14: Representative prompts and final verified code were documented by the authors. Available upon request from the corresponding author |
